# Vulvar melanoma: report on eleven cases and review of the literature

**DOI:** 10.1590/S1516-31802010000100008

**Published:** 2010-01-07

**Authors:** Glauco Baiocchi, João Pedreira Duprat, Rogerio Izar Neves, Elza Mieko Fukazawa, Gilles Landman, Gustavo Cardoso Guimarães, Leonardo Jacomo Valadares

**Affiliations:** I Brazil MD, MSc. Director, Department of Gynecology, Hospital do Cancer A. C. Camargo, São Paulo, Brazil.; II Brazil MD, PhD. Director, Department of Cutaneous Oncology, Hospital do Cancer A. C. Camargo, São Paulo, Brazil.; III United States MD, PhD. Associate professor, Department of Plastic Surgery, Pennsylvania State University, Pennsylvania, United States.; IV MD, MSc. Gynecological oncologist, Department of Gynecology, Hospital do Cancer A. C. Camargo, Sao Paulo, Brazil.; V MD, PhD. Pathologist, Department of Pathology, Hospital do Cancer A. C. Camargo, São Paulo, Brazil.; VI MD, MSc. Surgical oncologist, Department of Pelvic Surgery, Hospital do Cancer A. C. Camargo, São Paulo, Brazil.; VII MD. Surgical oncologist, Department of Cutaneous Oncology, Instituto Brasileiro de Controle do Cancer (IBCC), São Paulo, Brazil.

**Keywords:** Vulvar neoplasms, Lymphadenectomy, Sentinel lymph node biopsy, Review literature as topic, Melanoma, Neoplasias vulvares, Excisão de linfonodo, Biópsia de linfonodo sentinela, Literatura de revisão como assunto, Melanoma

## Abstract

**Context and Objective:**

Vulvar melanoma is a rare disease. We describe the experience of a single institution and review the literature.

**Design and Setting:**

Retrospective study at the Department of Gynecology, Hospital do Cancer A. C. Camargo.

**Methods:**

Eleven patients with vulvar melanoma attended between January 1987 and December 2006 were reviewed regarding clinicopathological characteristics, surgical therapy and follow-up.

**Results:**

The initial symptoms were vulvar lesions, pruritus, pain and bleeding. The median age was 64.8 years. The median depth of invasion was 3.08 mm. The staging ranged from IB to IIIC (American Joint Committee on Cancer, 2002). All the patients underwent vulvectomy. Two patients did not undergo primary elective lymphadenectomy. Bilateral inguinal lymphadenectomy was performed on five patients, and one had unilateral inguinal lymphadenectomy. Sentinel lymph node investigation was performed on three patients. Five patients had locoregional recurrence. Prolonged survival was only achieved in the absence of lymph node involvement. The median follow-up was 56 months. The median disease-free survival was 15 months and the median overall survival was 29 months.

**Conclusions:**

The prognosis for patients with vulvar melanoma is generally poor, with a high tendency towards regional and distant recurrence. Depth of invasion and lymph node involvement are the most important prognostic factors. In most cases, resection of the lesion with adequate margins may replace vulvectomy. Elective inguinal femoral lymphadenectomy remains the standard lymph node staging procedure. Sentinel lymph node investigation is feasible and should be performed by a multidisciplinary team with experience of this method.

## RESUMO

**Contexto e Objetivo:**

Melanoma de vulva é uma doença rara. Descrevemos a experiência de uma instituição e revisamos a literatura.

**Tipo de Estudo e Local:**

Estudo retrospectivo no Departamento de Ginecologia do Hospital do Câncer A. C. Camargo.

**Métodos:**

De Janeiro de 1987 a Dezembro de 2006, foram revisados aspectos clínico-patológicos, tratamento cirúrgico e acompanhamento de 11 pacientes com melanoma de vulva.

**Resultados:**

Lesão vulvar, prurido, dor e sangramento foram sintomas iniciais. A idade mediana foi 64,8 anos. A mediana da profundidade de invasão foi 3.08 mm. O estadiamento variou de IB a IIIC (American Joint Committee on Cancer, 2002). Todas as pacientes foram submetidas a vulvectomia. Duas pacientes não foram submetidas a linfadenectomia eletiva primária. A linfadenectomia inguinal bilateral foi realizada em cinco pacientes e uma foi submetida à linfadenectomia inguinal unilateral. A pesquisa do linfonodo sentinela foi realizada em três casos. Cinco tiveram recidiva locorregional. A sobrevida prolongada esteve relacionada com a ausência de comprometimento linfonodal. O tempo mediano de acompanhamento foi de 56 meses. A sobrevida mediana livre de doença foi de 15 meses e a sobrevida mediana global de 29 meses.

**Conclusões:**

O prognóstico das pacientes com melanoma de vulva geralmente é ruim, com tendência a recorrência regional e à distância. A profundidade de invasão e envolvimento linfonodal são os principais fatores prognósticos. Na maioria dos casos a ressecção da lesão com margens adequadas pode substituir a vulvectomia. A linfadenectomia inguino-femoral eletiva ainda é o procedimento padrão para estadiamento linfonodal. Pesquisa do linfonodo sentinela é factível e deve ser realizada por equipe multidisciplinar com experiência no método.

## INTRODUCTION

Vulvar melanoma is an unusual disease and accounts for 7 to 10% of all vulvar malignancies.^1,2^ It has an estimated annual rate of one case per 1,000,000 women.^3^ The incidence of vulvar melanoma that occurs in areas not exposed to ultraviolet radiation seems to have been stable over the years.^4^

Historically, radical vulvectomy and bilateral inguinal lymph node dissection were the recommended treatment for malignant vulvar melanoma, regardless of lesion size, thickness or depth of invasion.^5,6^ However, over the last few years, there have been some reports suggesting that acceptable survival has been achieved through less radical procedures performed on early disease.^6-14^

In the literature, it is suggested that the clinical and postoperative staging is the most significant predictor of survival. Depth of invasion and presence of ulceration^13,15-17^ are the most important histopathological prognostic factors.

## OBJECTIVE

The present study was designed to investigate clinical-pathological features, treatment data and patterns of recurrence and follow-up among the patients with vulvar melanoma treated in our institution. We also reviewed the current literature.

## MATERIALS AND METHODS

From January 1987 to December 2006, eleven patients with primary melanoma of the vulva were admitted to A. C. Camargo Cancer Hospital. The patients’ records were reviewed and all patients were staged in accordance with the American Joint Committee on Cancer (AJCC) Staging 2002.^18^ Clinical characteristics, histopathology, surgical therapy and follow-up were analyzed.

## RESULTS

The median follow-up time was 56.2 months. At the time of diagnosis, the median age was 64.8 years (range: 52-80). Detection of a vulvar lesion, pruritus, pain and bleeding were generally the initial symptoms. Three patients had no symptoms and the tumor was detected during routine medical examination.

The clinical and pathological characteristics, surgical data and clinical outcomes are summarized in Table 1.

**Table 1.** Clinical and pathological characteristics, surgical data and clinical course of the 11 patients included in this study

Patient	Age (years)	Staging (AJCC 2002)	Depth (mm)	Vulvar Surgery	Inguinal treatment	Local recurrence	Time until recurrence (months)	Follow-up (months)	Status
1	63	IIB	2.3	Vulvectomy, distal urethrectomy and colpectomy	Sentinel node (negative)	Urethra Inguinal contralateral	2 11	24	DFD*
2	55	IB	0.92	Vulvectomy	BIL†	–	–	103	AWD‡
3	52	IIB	2.2	Vulvectomy	UIL§	–	28	25	DFD*
4	67	IIC	5.0	Vulvectomy	–	Inguinal	4	20	DFD*
5	75	IIIC	3.0	Vulvectomy	BIL†	||	||	||	||
6	62	IIC	4.6	Vulvectomy	BIL†	Iliac	15	24	DFD*
7	72	IIC	4.3	Vulvectomy	–	Inguinal	13	47	DFD*
8	80	IIB	2.5	Vulvectomy	BIL†	–	44	44	DOC¶
9	63	IIIB	2.5	Vulvectomy	Sentinel node (positive)	Inguinal contralateral	8	14	DFD*
10	55	**	**	Vulvectomy	BIL†	–	240	240	AWD‡
11	69	IIIB	3.5	Vulvectomy	Sentinel node (positive)	–	20	20	AWD‡

* DFD = death from disease;

† BIL = bilateral inguinal lymphadenectomy;

‡ AWD = alive without disease;

§ UIL = unilateral inguinal lymphadenectomy;

|| Lost from follow-up;

¶ DOC = death from other causes;

** Initial excisional biopsy in other institution; AJCC = American Joint Committee on Cancer.

The median depth of invasion was 3.08 mm (range: 0.92-5.0) and ulceration was detected in eight patients. None of the patients had a family history of melanoma, but one patient had previously been treated for cutaneous melanoma. None of the cases presented with distant metastases at the time of diagnosis. The staging ranged from IB to IIIC (AJCC 2002).^18^

All the patients underwent radical vulvectomy. In one patient, the resection was extended to the distal urethra and vagina. Bilateral inguinal lymphadenectomy was performed on five patients. One patient with a unilateral tumor underwent ipsilateral inguinal lymphadenectomy. The sentinel lymph node procedure was performed using isosulfan blue dye and technetium sulfur colloid for three patients. None of the patients received adjuvant radiation or immunotherapy.

Five patients underwent another surgical procedure, which was performed after local or regional recurrence. One patient developed a melanoma *in situ* in the perineum that was considered to be another primary tumor.

Vulvectomy without lymphadenectomy as the first treatment was performed for two patients. Both of these patients had tumor thicknesses greater than 4 mm, and they had recurrence in the groin after four and 13 months of follow-up. Both died due to distant recurrence, even though inguinal femoral lymphadenectomy had been performed. There was one other patient with tumor thickness greater than 4 mm. She underwent radical vulvectomy with negative bilateral inguinal lymphadenectomy as the first treatment, but after 15 months of follow-up, she presented pelvic lymph node, bone and pulmonary recurrences.

Three patients underwent the sentinel node procedure. The first had a negative unilateral sentinel node, but presented with a contralateral metastatic lymph node after 11 months of follow-up. The second had a micrometastatic unilateral sentinel node. She underwent unilateral inguinal femoral lymphadenectomy that resulted in a residual metastatic lymph node. Subsequently, she also presented recurrence in the contralateral groin and simultaneous vagina and lung recurrence after seven months of follow-up. The third patient presented a micrometastatic unilateral sentinel node and a negative contralateral sentinel node. Unilateral inguinal femoral lymphadenectomy resulted in absence of metastatic nodes. She remained free of disease after 20 months of follow-up. All of these three patients had tumor thickness of less than 4 mm.

One patient had synchronic endometrioid endometrial cancer (Stage IBG2 of the International Federation of Gynecology and Obstetrics [Fédération Internationale de Gynécologie et d’Obstétrique, FIGO], 1988) and vulvar Paget’s disease. Total abdominal hysterectomy, bilateral salpingoophorectomy, omentectomy and pelvic lymphadenectomy for the uterine cancer and vulvectomy were performed during the same surgical procedure.

All the patients who developed distant metastases died from the disease. A single patient was treated with chemotherapy (fotemustine and decarbazine) and immunotherapy (interferon), but the response was poor and the patient only survived for nine months.

One patient was lost from the follow-up. Six patients died from the disease. One patient died due to another cause, after 44 months of follow-up.

Three of the patients are still alive and disease-free with follow-ups of 20, 103 and 238 months. Prolonged survival was only achieved for patients with no lymph node involvement. On the other hand, one patient with no metastatic lymph nodes presented a distant relapse.

The median overall length of survival was 29.3 months. The median disease-free length of survival was 15 months and the mean length of time from the diagnosis of the first recurrence to death was 14.8 months.

A systematic review of the following databases was performed: PubMed, Cochrane Library, Lilacs (Literatura Latino Americana e do Caribe em Ciências da Saúde) and SciELO (Scientific Electronic Library Online). The search strategies and results obtained are shown in Table 2.

**Table 2.** Systematic review of the literature

Database	Search strategy	Results
PubMed	Vulvar neoplasms [MeSH] AND Melanoma [MeSH]	102 articles
		48 case reports
		24 review articles
		27 case series
		2 cross-sectional prevalence studies
		1 clinical trial
Lilacs	Vulvar neoplasms [MeSH] AND Melanoma [MeSH]	10 articles
	Neoplasias vulvares [DeCS] AND Melanoma [DeCS]	5 case reports
		4 case series
		1 review article
Cochrane	Vulvar neoplasms [MeSH] AND Melanoma [MeSH]	no article
SciELO	Neoplasias vulvares [DeCS] AND Melanoma [DeCS]	no article

MeSH = Medical Subject Headings; DeCS = Descritores em Ciências da Saúde; Lilacs = Literatura Latino-Americana e do Caribe em Ciências da Saúde; SciELO = Scientific Electronic Library Online.

## DISCUSSION

Malignant melanoma of the vulva is the second most common malignancy of the vulva, but accounts for only 5 to 11% of all melanoma cases.^5^ Consequently, most reports are limited by consisting only of small retrospective numbers of cases over a long period of time. Over recent decades, few studies reviewing more than 40 patients have been published.^2,5,6,10,14,16,17,19^ In 1994, Phillips et al.^13^ published the only prospective study and, in 1999, Creasman et al.^15^ published the largest report of 569 cases from the National Cancer Data Base.

The disease is usually diagnosed in the sixth and seventh decades of life and the initial symptoms are generally vulvar lesions, pruritus or bleeding.^5^

The biological behavior of primary vulvar and extragenital cutaneous malignant melanoma appeared to be comparable.^13,16^ However, some studies^1,3,20^ have shown that the overall prognosis for patients with vulvar melanoma is generally poor, compared with cases of cutaneous melanoma and invasive vulvar carcinoma, with a higher tendency towards regional and distant recurrence.

The primary tumor resection procedure, the efficacy of regional node dissection and the role of sentinel node biopsy are still unresolved issues. Some authors have recommended radical local excision with adequate margins, because they noted that radical vulvectomy does not provide any survival advantage,^7-11^ while causing worse impairment of body image.

There are few published papers on sentinel lymph nodes in relation to vulvar melanoma and there is insufficient evidence for their routine use.^21-24^ In 2002, de Hullu et al.^21^ reported his preliminary experience from nine patients who underwent sentinel node mapping for vulvar melanoma. The sentinel node was identified in all patients and three patients presented positive sentinel nodes. Two of those patients presented groin recurrences within the first 12 months of follow-up. Both had thicknesses of more than 4 mm and negative sentinel nodes. Subsequently, four other studies including 17 cases of vulvar melanomas were published.^19,22-24^ The groin detection rate ranged from 83 to 100%.

The sentinel lymph node procedure was introduced in our institution for cutaneous melanoma in 1998. In our study, the sentinel node procedure failed to predict the inguinal lymph node status in one of three patients. Two patients had relapses in the contralateral groin. One patient had a false negative sentinel lymph node, probably related to a local advanced primary tumor that invaded the distal urethra and distal vagina. The other patient had two positive unilateral lymph nodes and presented recurrence in the contralateral groin, which previously had not shown any sentinel node.

Five out of 10 patients presented locoregional recurrence and all of these cases were related to groin lymph nodes. We also found a 60% distant recurrence rate in our series. After distant recurrence was detected, the survival was of short duration, ranging from 1.3 to 9.2 months (mean: 5.2).

The five-year survival rates for vulvar melanoma reported in the literature range from 20 to 56%.^2,5,8,10^ Involvement of regional lymph nodes, depth of invasion and presence of ulceration are widely accepted prognostic factors.^25^ Our study confirms the overall poor prognosis for vulvar melanoma and, as noted by Jaramillo et al.,^7^ none of the patients with confirmed metastatic lymph nodes belonged to the group of long-term survivors.

## CONCLUSIONS

Based on our report and review of the literature, primary tumor resection should be performed in accordance with the accepted standards for cutaneous melanoma. In most cases, wide excision with 2-3 cm margins may replace radical vulvectomy. The lymph node status is the most important prognostic factor. Elective inguinal femoral bilateral lymphadenectomy is still the standard lymph node staging procedure, but there is no evidence that it has a therapeutic role or any impact on overall survival. The sentinel lymph node procedure is a feasible method and should be performed by a skilled multidisciplinary team on well-selected patients, in the absence of a clinically detected metastatic lymph node.
